# Hyperthermia as a trigger for Takotsubo syndrome in a rat model

**DOI:** 10.3389/fcvm.2022.869585

**Published:** 2022-07-26

**Authors:** Matthew H. Tranter, Bjorn Redfors, Peter T. Wright, Liam S. Couch, Alexander R. Lyon, Elmir Omerovic, Sian E. Harding

**Affiliations:** ^1^Faculty of Medicine, Imperial College London, Hammersmith Campus, National Heart and Lung Institute (NHLI), London, United Kingdom; ^2^Oriel College, University of Oxford, Oxford, United Kingdom; ^3^Department of Molecular and Clinical Medicine/Cardiology, Sahlgrenska Academy, University of Gothenburg, Gothenburg, Sweden; ^4^School of Life and Health Sciences, University of Roehampton, London, United Kingdom

**Keywords:** Takotsubo, stress, hyperthermia, catecholamine, isoprenaline

## Abstract

Takotsubo syndrome is a well-characterized cause of acute yet reversible heart failure associated with periods of intense emotional stress, often mimicking on presentation an acute coronary syndrome. Animal models of Takotsubo syndrome have been developed, either through the application of a stressor, or administration of exogenous catecholamine. We found that in a model of isoproterenol-induced Takotsubo syndrome in anesthetized rats hyperthermia (40–41°C) would occur after the administration of isoproterenol. Maintenance of this hyperthermia would result in an apical hypocontractility typical of the syndrome, whereas prevention of hyperthermia with active cooling to maintain a euthermic core body temperature prevented (but did not subsequently reverse) apical hypocontractility. *In vitro* experimentation with isolated cardiomyocytes showed no effect of hyperthermia on either baseline contractility or contractility change after beta-adrenoceptor stimulation. We suggest that the rise in body temperature that is characteristic of catecholamine storm may be a component in the development of Takotsubo syndrome.

## Introduction

Takotsubo syndrome (TTS), previously known as Takotsubo cardiomyopathy or stress cardiomyopathy, is an acute and reversible heart failure that typically occurs after a period of intense emotional or physical stress; symptoms include chest pain and dyspnea and, as a result, can mimic an acute coronary syndrome (1). However, no culprit macrovascular pathology can be found on coronary angiography and a diagnosis of TTS is made due to the observation of the characteristic hypocontractile apex coupled to a hypercontractile base, although variants on this pattern have been observed, e.g., midventricular or basal hypocontractility and also right ventricular involvement (2, 3).

A number of models have been developed to study TTS, including rat *in vivo* and cellular models (4–6), and human induced pluripotent stem cells from Takotsubo syndrome patients (7), as well as healthy controls (8). These have produced insights on the various phases of Takotsubo syndrome, from the very acute response which includes adrenaline surge, lipotoxicity, nitric oxide generation [and a potential increase in nitrosative stress (9)] and endothelial damage, to the long-term effects through maintained inflammatory damage (10). Many models consist of the exogenous administration of catecholamines, such as the recently described induction of TTS-like contractile dysfunction using intraperitoneal (IP) 50 mg.kg^−1^ isoproterenol (5). We attempted to reproduce this model in our laboratory as the time course of dysfunction is similar to the human disease course, and with a prolonged period of apical hypokinesia there would be scope to test therapies during the establishment of TTS-like contractility rather than having to administer them before (4). However, the dysfunction observed in (5) could not be recreated in our laboratory.

It was noticed during initial experiments that body temperature tended to rise after isoprenaline administration. A rise in body temperature is known to be produced by high catecholamine levels (once known as “emotional fever”) (11), as is a suite of immunoinflammatory changes (12). In our experiments, the rise in body temperature was controlled by adjusting the external heating support for the anesthetized animal, which remained euthermic throughout. After discussion with the Omerovic group at the University of Gothenberg (the authors of the original IP isoprenaline study) a study was carried out to determine whether allowing this temperature rise in a controlled manner would also allow the apical dysfunction characteristic of TTS to occur. We found that hyperthermia was an essential component in the induction of TTS in this rat model, yet the removal of the hyperthermia after the initial induction phase did not change outcome. This may have relevance to the circumstances leading to the clinical presentation of Takotusbo syndrome.

## Methods

All studies complied with Animals (Scientific Procedures) Act (1986), European Directive 2010/63/EU and with the 8th edition of the *Guide for the Care and Use of Laboratory Animals* published by the US National Institutes of Health. Animals were housed in groups of four and experiences a 12 h light/dark cycle. All rats were male and of the Sprague Dawley strain and obtained from Charles River Laboratories (UK). All animals were killed at the end of the procedure by an approved Schedule 1 method.

### Induction of experimental Takotsubo syndrome model

Rats were anesthetized using IP ketamine-midazolam (50 and 5 mg.kg^−1^, respectively) with 25/2.5 mg.kg^−1^ administered PRN to maintain a sufficient plane of anesthesia for consistent echocardiographic measurement. 50 mg.kg^−1^ (-)-isoproterenol was administered IP and rats were monitored by two-dimensional parasternal long axis echocardiography (Visualsonics Vevo 770), with M-mode echocardiography used to assess regional cardiac function, compared to a baseline reading taken before catecholamine administration. A lead II ECG was taken using subdermal electrodes connected *via* a BioAmp to a PowerLab data acquisition system (AD Instruments).

### Body temperature manipulation

**Figure 1:**
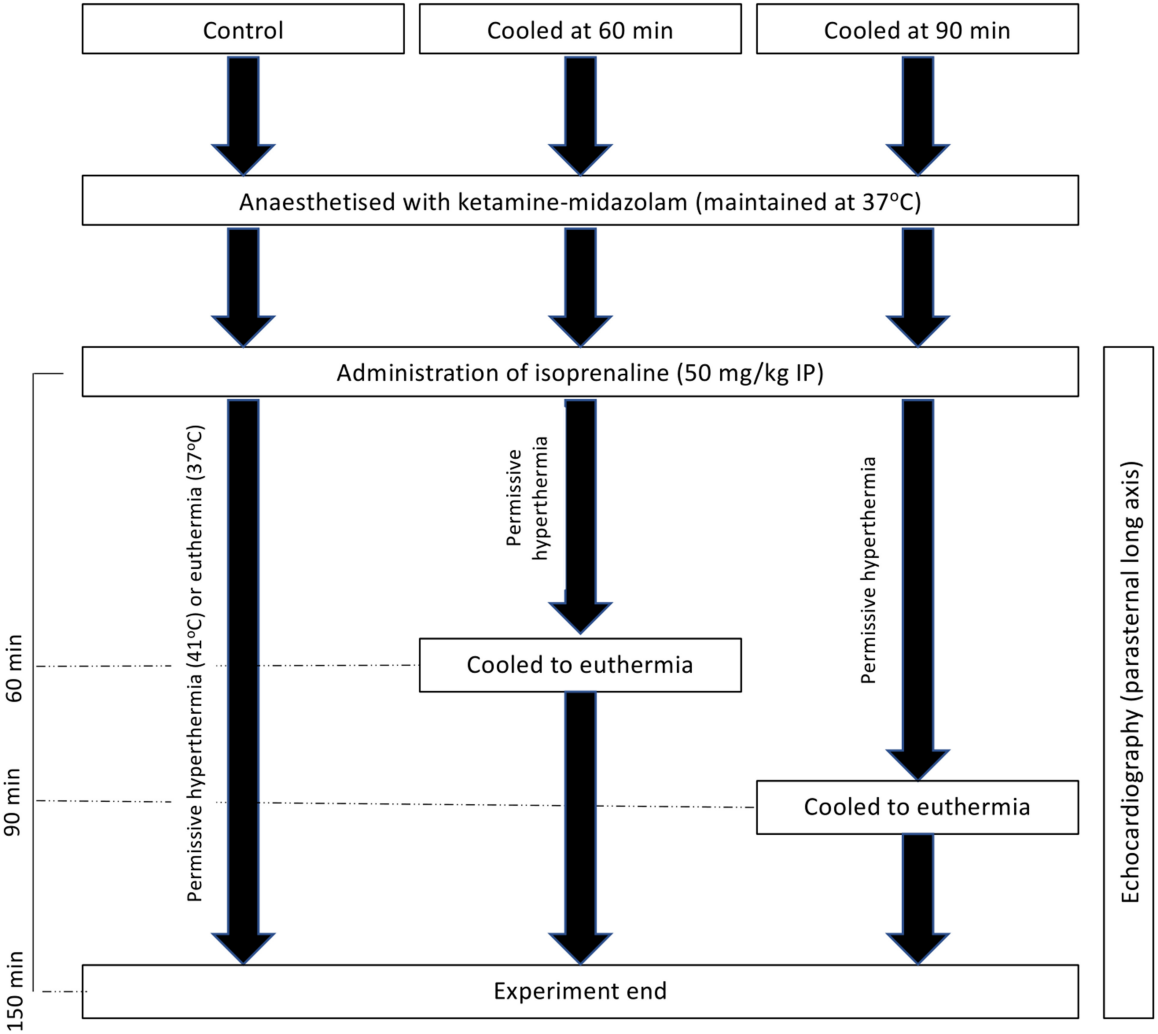
Experimental schematic of hyperthermic experiments with and without cooling to 150 min post-isoprenaline administration.

Before catecholamine administration, body temperature was maintained between 37 and 38°C using a homeothermic temperature monitor, rectal temperature probe, and heating blanket (Harvard Apparatus). Heating was maintained at a similar level after the administration of isoprenaline, with a new set-point of 41°C used. Cooling, if required at either 60 or 90 min post-isoproterenol administration (Figure 1), was achieved through the removal of external heat sources and the application of ice packs to the abdomen until the rectal temperature was back in the euthermic zone, after which external heating was reapplied as required to maintain euthermia. For experiments shown in the Supplementary Figures, male rats were randomized to either of two groups and subsequently received 50 mg/kg isoprenaline intraperitoneally. Body temperature was maintained at 37.5 ± 0.5°C (normothermia) for the duration of the experiments in the first group whereas in the second group body temperature was first allowed to rise spontaneously and, starting at 15 min post isoprenaline, was maintained at 41 ± 0.5°C (hyperthermia) for the remainder of the experiment. Extent of akinesia was traced in the long axis and expressed as percentage of total LV endocardial length.

### Rat cardiomyocyte isolation and *in vitro* contractility studies

Cardiomyocytes were isolated from adult male rats as previously described using a two-enzyme technique (13) and studied using a video edge detection and tracking system (IonOptix) as previously described (4). Cardiomyocytes were paced at 0.5 Hz throughout all studies and contraction was determined as shortening relative to the diastolic length. For studies assessing changes in baseline (i.e., without catecholamines present), cells were perfused with Krebs-Henseleit (KH) solution (1 mM Ca^2+^) at 37°C for 10 min, after which the temperature either remained constant or was elevated to 41°C. Contractility was measured at 10 and 20 min post-temperature change and changes are expressed relative to contractility at the end of the 10 min baseline period. For isoproterenol hyperthermia studies, cells were perfused with KH at either 37 or 41°C for 10 min, at the end of which a baseline recording was taken. 10^−6^ M isoproterenol was then perfused and contractility measurements taken at 10 and 20 min post-isoproterenol, and expressed as a relative change in contractility compared to the end of the 10 min baseline.

### Statistical analysis

All *in vivo* contractility and temperature data was analyzed using two-way repeated measures ANOVA calculations with either Tukey's (for whole experiment analysis) or Dunnett's (individual time-point analysis) *post-hoc* tests for comparison to baseline or apex vs. base. One-way ANOVA with Sidak's *post-hoc* test was used to compare time-points with baseline for body temperature changes, and for cellular contractility studies. Statistical significance was defined as *P* < 0.05 and data was tested for normality using a Komogorov–Smirnov test. All statistical analyses were carried out using GraphPad Prism 6 and data is presented at Mean ± SEM unless otherwise stated. Intra-experimental mortality was not observed in any study and therefore not presented graphically.

## Results

### 50 mg.kg^–1^ isoproterenol at euthermic temperatures does not cause a recapitulation of the Takotsubo-like contractility pattern

**Figure 2:**
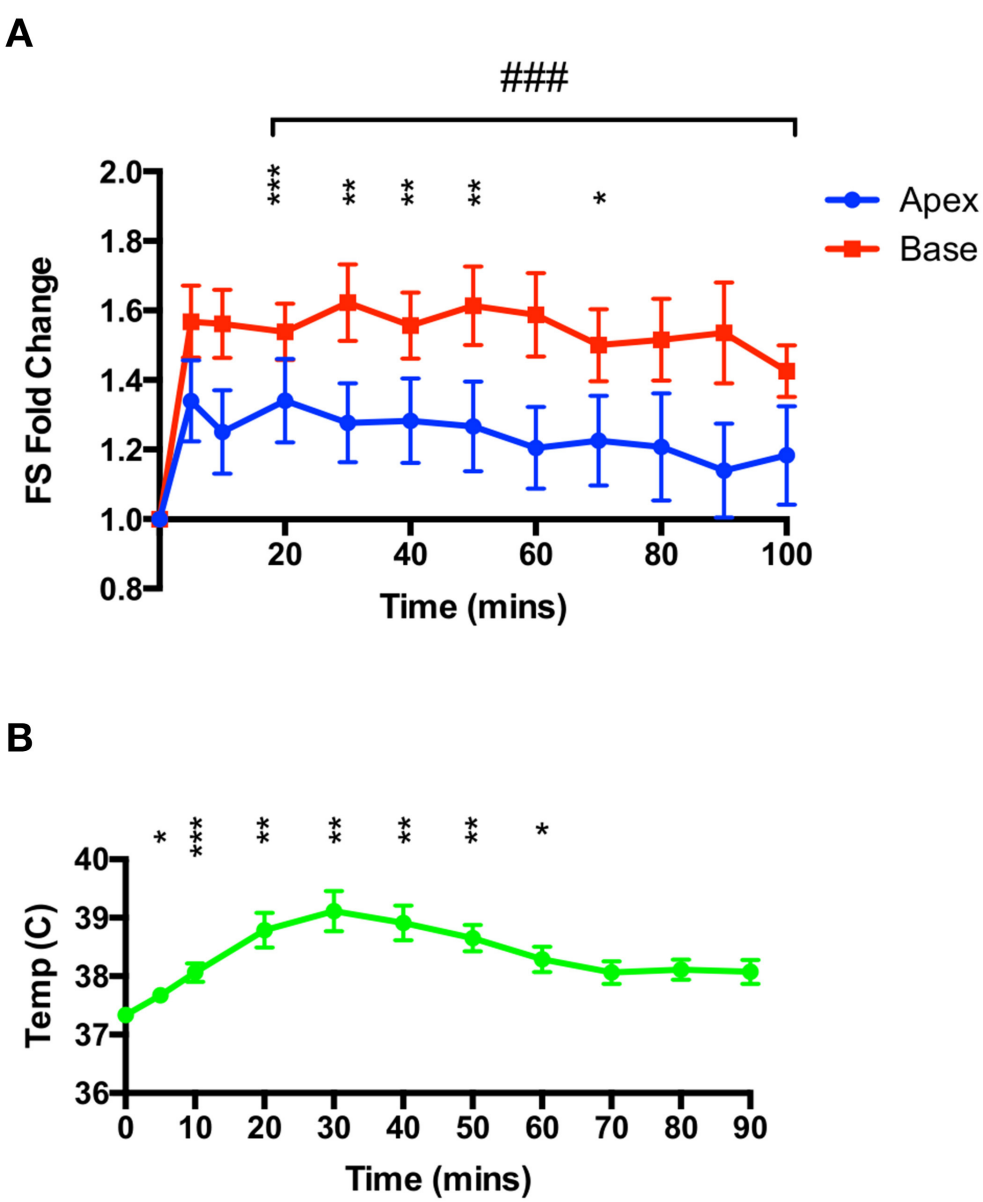
High-dose isoproterenol (50 mg.kg^−1^) under euthermic conditions does not result in TTS-like contractility. **(A)** 50 mg.kg^−1^ isoprenaline under ketamine-midazolam anesthesia resulted in a significant positive inotropic response in both apical and basal myocardial segments, although not for all time-points in the apex. No significant difference was seen between apical and basal contractility (*n* = 8). *P < 0.05, **P < 0.01, ***P < 0.001 vs. baseline (apex); ^###^*P* < 0.001 vs. baseline (base) two-way repeated measures ANOVA. **(B)** Rectal temperature increases after isoproterenol, although this is attenuated by removing heating from the animal. *P < 0.05, **P < 0.01, ***P < 0.001 relative to time 0, one-way ANOVA (*n* = 8 animals).

Rats were injected with 50 mg.kg^−1^ IP while under constant ketamine-midazolam anesthesia and serial M-mode measurements of apical and basal segment contractility were taken. Over the 100 min period following isoproterenol administration, no overall apical hypocontractility was seen, but rather a mild and sustained positive inotropy in both segments (Figure 2A). However, no information on right ventricular function was gathered. A mild yet significant increase in body temperature was seen (Figure 2B), which was attenuated by removing all heating and applying cooling as needed.

### TTS-like contractility can be recapitulated with a 50 mg.kg^–1^ isoproterenol dose when the hyperthermic response can manifest

**Figure 3:**
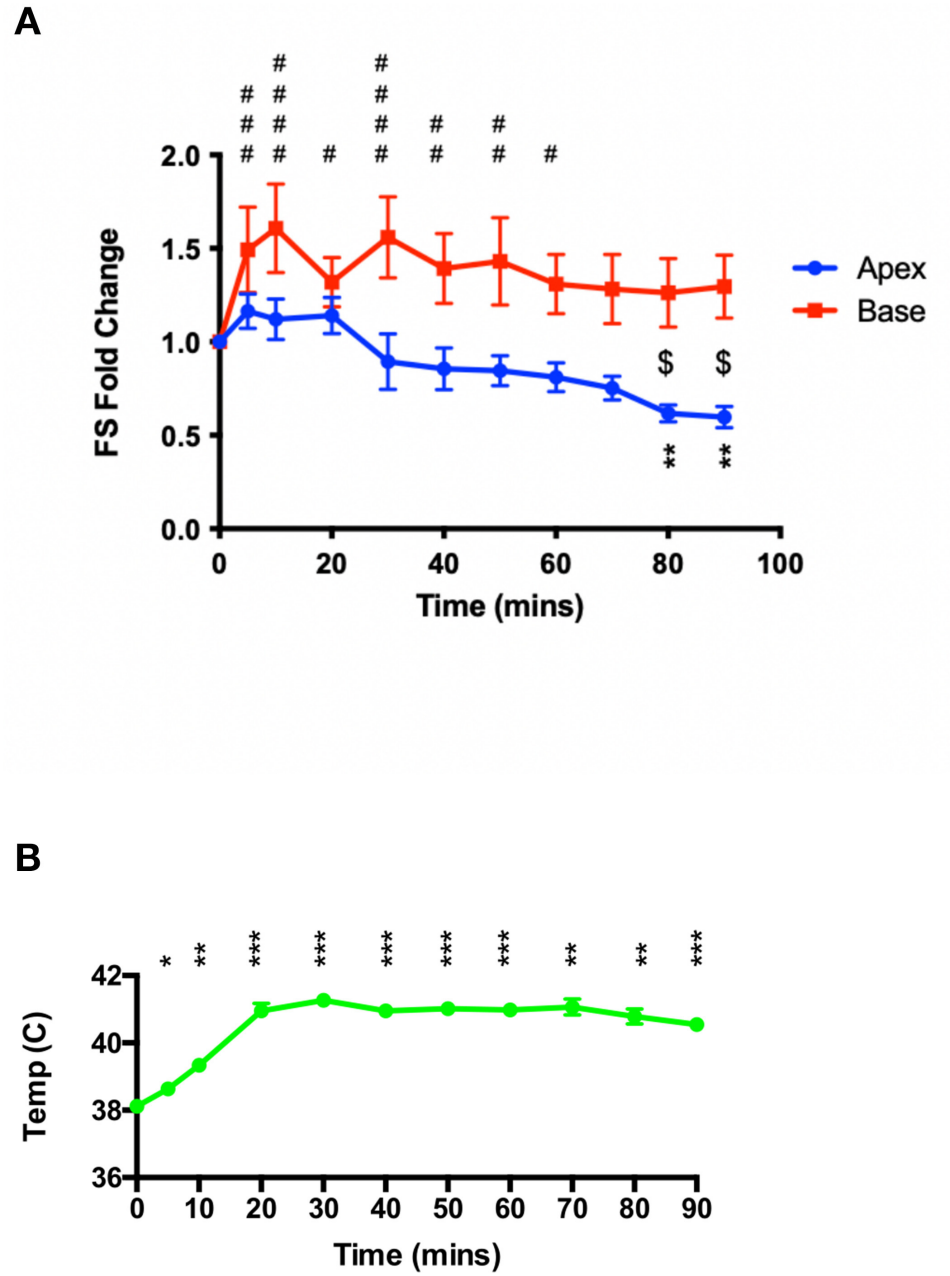
High-dose isoproterenol (50 mg.kg^−1^) administration under hyperthermic conditions (~41°C) results in apical-hypocontractility. **(A)** 50 mg.kg^−1^ isoprenaline under ketamine-midazolam anesthesia resulted a significant positive inotropic response was seen in the basal myocardial segment for the first 60 min, whereas the apical myocardial segment showed a significant negative inotropic response at 80 and 90 min post-isoprenaline. **P < 0.01 apex vs. baseline; ^$^*P* < 0.05 apex vs. base; ^#^*P* < 0.05, ^##^*P* < 0.01, ^###^*P* < 0.001, ^####^*P* < 0.0001 base vs. baseline, two-way repeated measures ANOVA (*n* = 6). **(B)** Sustained heating of the animal maintains a significant elevated body temperature post-isoproterenol. *P < 0.05, **P < 0.01, ***P < 0.001 relative to baseline temperature, one-way ANOVA (*n* = 6 animals).

As shown in Figure 2, 50 mg.kg^−1^ isoproterenol, when administered under ketamine-midazolam anesthesia, causes a hyperthermia response that was, in initial studies, attenuated to maintain a euthermic temperature. In a second set of studies, animals were allowed to reach 41°C and maintained at that temperature throughout the 90 min of serial echocardiographic recording post-isoproterenol. This resulted in a negative inotropy in the apex at the 80 and 90 min timepoints compared to baseline, and a significant difference in inotropic change between apex and base at the 80 and 90 min timepoints (Figure 3). Heart rates for these animals can be found in the Supplementary Data.

### Cooling animals back to euthermia after the initial induction phase does not reverse or attenuate the apical dysfunction caused by high dose isoproterenol

**Figure 4:**
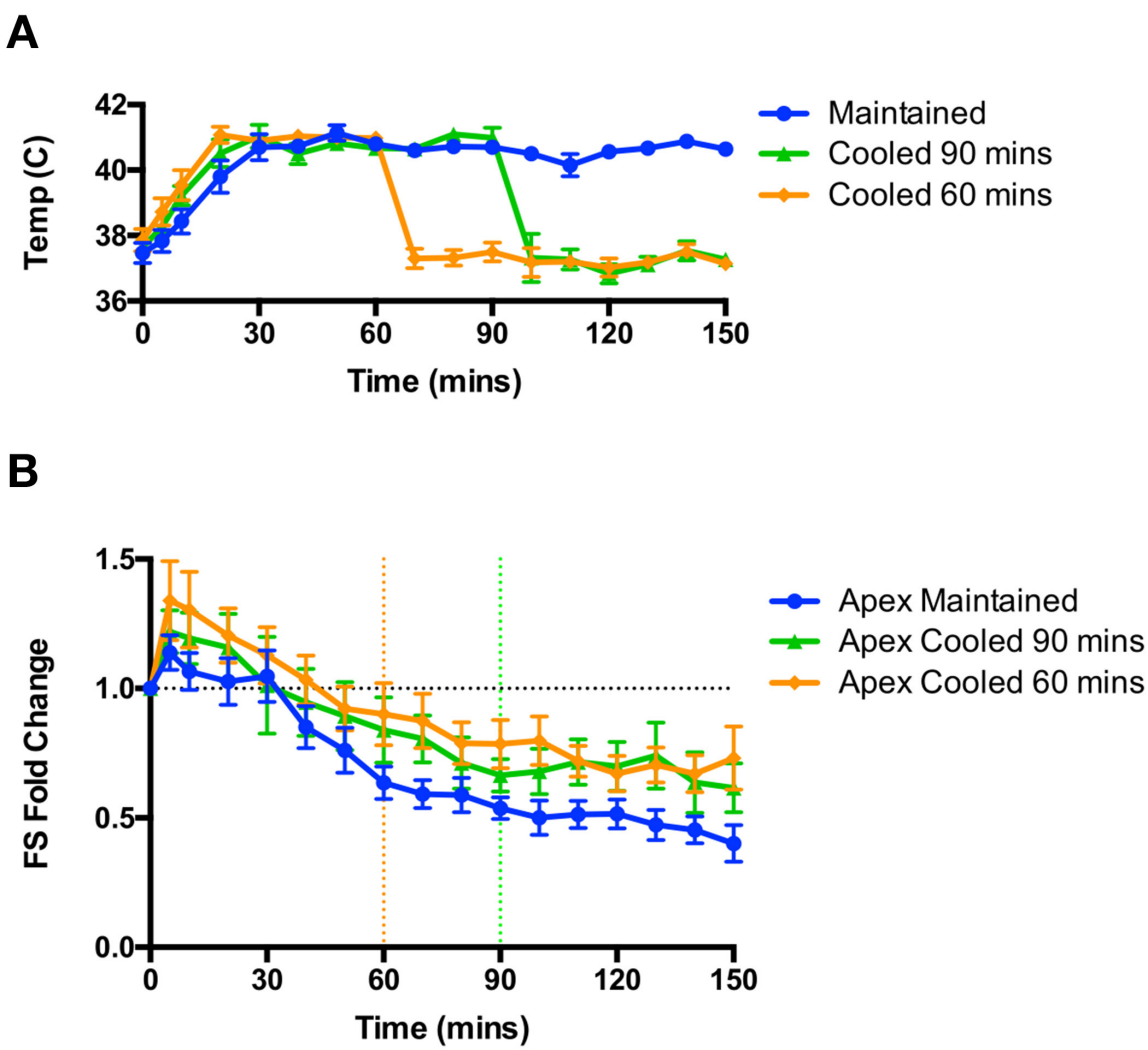
Rapid cooling of animals after the induction of apical dysfunction did not reverse or attenuate the hypocontractility. **(A)** Rapid changes in body temperature could be achieved through the application of cooling. **(B)** No effect of cooling was observed on apical dysfunction on rats cooled at 60 min (from orange dotted line) or 90 min (from green dotted line); *n* = 6 animals per group. Repeated measures ANOVA.

As hyperthermia was essential to the induction of apical dysfunction, we hypothesized that restoration of a euthermic body temperature would either attenuate or reverse the dysfunction. In a separate set of studies shown in Figure 4, rats were randomized prior to study either to be cooled at 60 or 90 min post-isoproterenol, or be maintained at 41°C; all animals were studied to 150 min post-isoproterenol regardless of group. Through the application of cooling packs we were able to rapidly reduce the body temperature back into the euthermic range (Figure 4A). We found no significant change in apical hypocontractility in either cooled group compared to the maintained group; apical hypocontractility was sustained in all three groups (Figure 4B).

### *In vitro* hyperthermia had no effect on baseline contractility or beta adrenoceptor responsiveness

**Figure 5:**
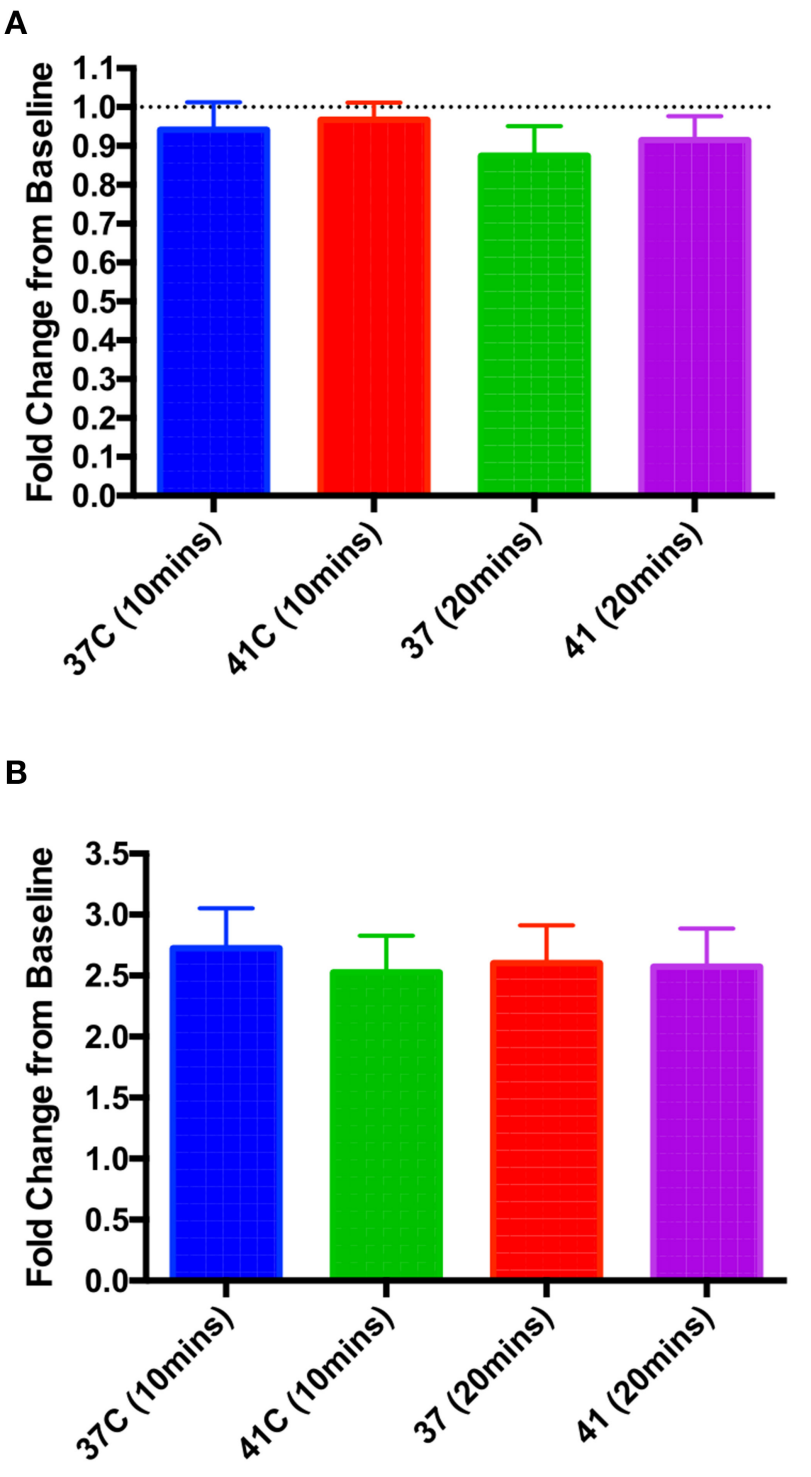
*In vitro* hyperthermia had no significant effect on either baseline contractility at 10 or 20 min post-temperature change **(A)** or the increase in contractility when 10^−6^ M isoproterenol was applied at either 10 or 20 min post-isoproterenol **(B)**. One-way ANOVA, *n* = 9 and 10 cells **(A)**, 8 and 7 **(B)**, 37 and 41°C, respectively.

Isolated rat left ventricular cardiomyocytes were perfused with Krebs-Henseleit (KH) solution at 37°C for 10 min, after which a measurement of cellular shortening was taken and perfusion temperature increased to either 41°C or maintained at 37°C (control). No significant changes in contractility were seen with this increase in temperature (Figure 5A). In a separate set of experiments, isolated cardiomyocytes were perfused with KH at either 37°C or 41°C, and after a 10 min stabilization period, a baseline measurement of cellular shortening was taken and isoproterenol (10^−6^ M) was added to the perfusate. Measurements were taken at 10 and 20 min post-isoproterenol addition, and no significant differences were seen between the two temperature groups at either time point (Figure 5B).

## Discussion

TTS is a syndrome well-associated with an excess of catecholamines (14), and therefore pre-clinical models using excess catecholamines are useful to delineate the steps the lead to the induction of TTS. In a previous model of TTS developed by the Imperial laboratory (4) a single IV dose to produce a high epinephrine level (approximately equivalent to an Epi-Pen in human) was sufficient to induce a transient apical dysfunction in male rats, where contractility was at a nadir at 20–25 min post-epinephrine and recovered at 60 min post-epinephrine. While this is important to study immediate effects of a catecholamine spike, there are longer-term changes triggered which may underlie a persistent cardiac damage. Models using an intraperitoneal injection of isoproterenol, where animals are studied over hours to days/weeks, have shown an activation of immune responses (12) and an upregulation of pathways involved in inflammation (15). However, while attempting to reproduce the well-validated model of Shao et al. (5) we experienced difficulties, which we resolved in a collaborative effort with the Omerovic laboratory.

**Figure 6:**
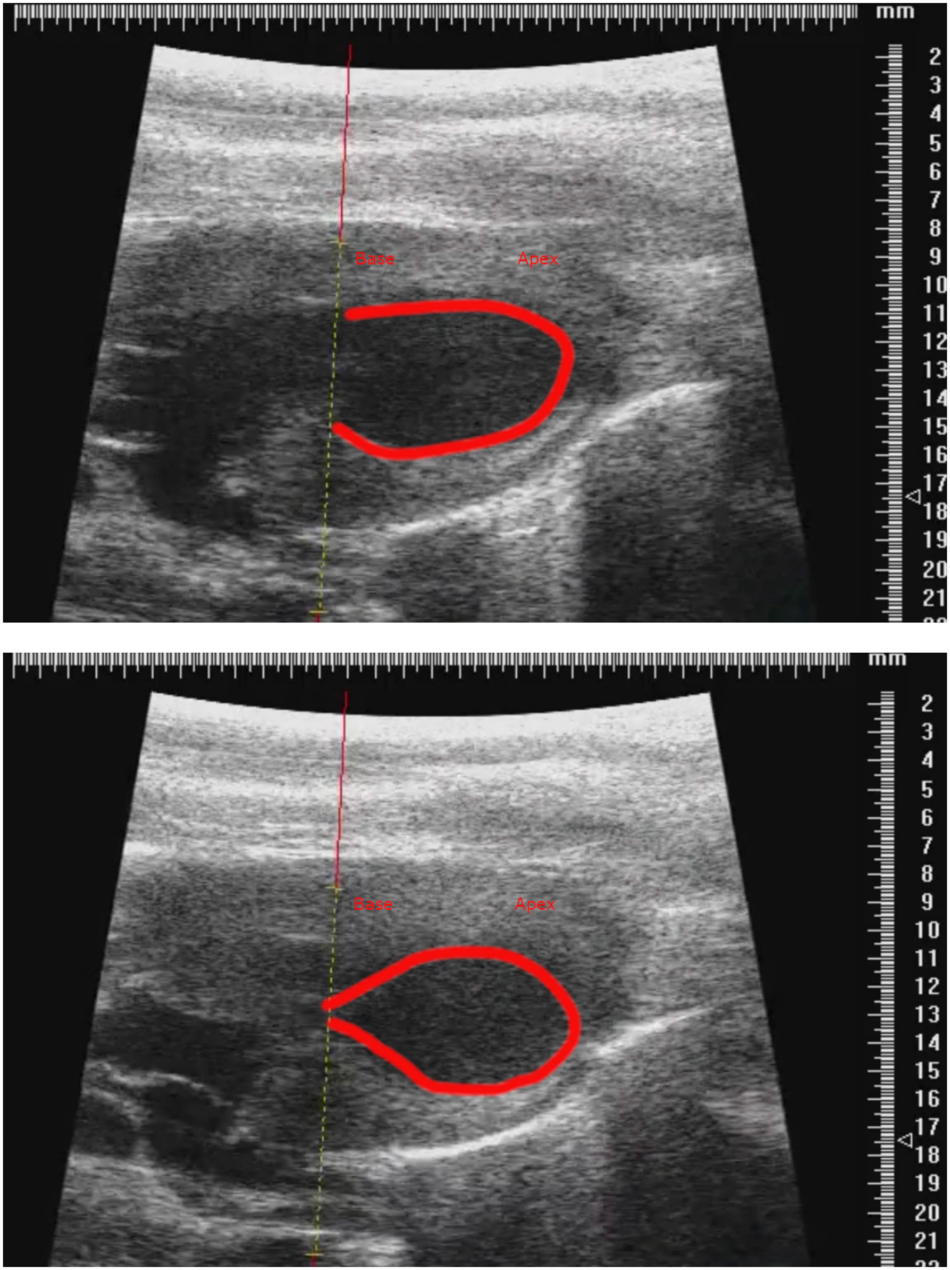
Representative parasternal long axis B-mode images of end-diastole **(top)** and end-systole **(bottom)** after induction of TTS-like contractility (base: left; apex: right), with trace (red line) drawn around endocardial border. Scale in mm.

We noticed that after isoprenaline administration, animals tended to increase their body temperature, and after discussion between the two groups we determined that allowing the development of the natural transient isoproterenol-induced hyperthermia was essential for the consistent induction of TTS-like contractility in this animal model (Figure 6). Groups wishing to replicate this TTS model should pay close attention to body temperature and allow moderate hyperthermia to occur.

There has been an anecdotal or correlative association between increased body temperature and Takotsubo syndrome in a number of circumstances. Takotsubo syndrome is statistically more likely to occur in summer or early autumn (unlike the winter presentation preference for most cardiac diseases) and is separately associated with higher ambient temperatures (16, 17). There has also been an afternoon predominance for TTS presentation compared to night-time (18) Fever and particularly sepsis have strong associations with Takotsubo syndrome (19), and high temperature was a predictor of worse outcomes in a series of 421 admissions for the syndrome (20). Hyperthyroidism, which is associated with raised body temperature, has been observed in a proportion of patients with Takotsubo syndrome (21). It is tempting to speculate that the temperature dysregulation and hot flushes in post-menopausal women may be an additional trigger for the syndrome. Furthermore, there have been increasing reports of TTS associated with COVID (22), and an early review has suggested that the morbidity and mortality of TTS may be exacerbated when superimposed on the hyper-inflammatory COVID background (23).

As hyperthermia was essential for the induction of TTS-like contractility changes, we sought to determine its importance in the maintenance of this contractility pattern. We found that cooling the animals back to a physiological body temperature at either 60 or 90 min post-isoproterenol had no effect on apical dysfunction, and changes in contraction followed a similar trend to animals maintained at 41°C. However, we do not know the effect of this cooling on long-term prognosis. Cellular experiments showed that hyperthermia alone did not increase contractility of cardiomyocytes, nor increase beta-adrenoceptor responsiveness, and therefore hyperthermia is affecting either the heart on an organ level, or at an integrated body level. The role of circulating immune cells was evident in the study on immune activation by single dose isoproterenol, and adoptive transfer of splenocytes from the isoproterenol-treated mice induced left ventricular dilation and impaired cardiac function in healthy recipients (12).

### Mechanism of hyperthermia in TTS induction

Hyperthermia causes a wide range of physiological responses at enzymatic, cellular, organ, and whole-body levels. Through both pre-clinical models and clinical evidence, a number of hypotheses for the pathophysiology of TTS have been proposed. These include microvascular dysfunction (24), lipotoxicity within cardiomyocytes (5), and beta-adrenoceptor stimulus trafficking at the level of the cardiomyocyte (4). It may be that hyperthermia is acting on these, or through other mechanisms to increase apical dysfunction: there are a number of candidate underlying pathways, and their actions may not be mutually exclusive.

Increases in body temperature from 38 to 41°C in rats have been shown to increase sympathetic nerve discharge rate (11, 25), and may be supporting the hyper-catecholaminergic state present in the rat when isoproterenol is administered, particularly given the half-life of isoprenaline is <5 minu and a single dose of isoprenaline has been shown to induce persistent pathological cardiac changes (12). AMP-activated protein kinase (AMPK) is ubiquitously expressed throughout the body, and has been implicated in the pathophysiology of TTS as endomyocardial tissue biopsies taken during the acute TTS phase show increased PI3K/Akt pathway activity (26), which can be activated by AMPK (27). Hyperthermia has been shown to activate AMPK in cancer cells (28), and hypothermia shown to inhibit AMPK in the brain (29). If AMPK is activated by hyperthermia in the setting of TTS, this could be contributing to the protective mechanisms thought to be present in this disease as AMPK activation has been shown to reduce cardiomyocyte apoptosis (30, 31). Heat shock proteins are also likely to be activated by this thermal stress (32), and have been shown to reduce both isoproterenol-induced cardiac apoptosis (33) and remodeling (34). One study has demonstrated the inhibition of the late-sodium currents by hyperthermia in iPS-derived cardiomyocytes (35), which interestingly was also seen in an iPS-derived cardiomyocyte model of TTS (8). We know that nitrosative stress may play a role in TTS (36, 37), which has been shown to be induced by hyperthermia (38). It has also been shown that alpha-1 adrenoceptor signaling *via* reactive oxidative stress increases the arrhythmogenicity of the myocardium during high levels of catecholamines (39), although in our study isoproterenol was used, which does not act as an agonist on alpha-1 adrenoceptors. Given the increased incidence of a prolonged QT interval in TTS in both models and patient cohort analysis (8, 40), the role of hyperthermia on reactive oxygen species generation and its arrhythmogenicity in the setting of excess catecholamines should be further assessed.

The role of hyperthermia directly on beta-adrenoceptors at a molecular level is poorly understood: one conference abstract has demonstrated reduced beta-adrenoceptor responsiveness in bladder detrusor muscle between 37 and 42°C (41), whereas many studies have been carried out on beta-adrenoceptor responses at hypothermic temperatures. In isolated rat cardiomyocytes, decreases in temperature to 15°C showed an increase in beta-adrenoceptor affinity (using radioactive-labeled propranolol) compared to normothermia (37°C). Hypothermia also does not prevent a beta-adrenoceptor response, as shown in a porcine model of hypothermia, where 30 ng/kg/min infusion of epinephrine resulted in a positive inotropic response at 32°C (42). However, in another experiment at 25°C, isoprenaline resulted in no positive inotropic response (43). Therefore, there is conflicting evidence with regards to catecholamine stimulation with varying body temperature, and further work to elucidate the molecular changes in this model of hyperthermia-facilitated TTS is required.

In a recent computational model of TTS a failure of the Frank-Starling mechanism was shown to increase apical ballooning at end systole (44). In a study that assessed the effects of hyperthermia on the Frank-Starling mechanism in anesthetized dogs, it was shown that hyperthermia of a similar range to the animals presented in this paper (40–41°C) resulted in a significant decrease in the gradient of inotropy change with respect to preload, thereby showing a failure of the Frank-Starling mechanism (45). This could also partly explain the lack of effect seen in isolated cardiomyocytes as they are unloaded. Although a tempting proposition, and one which certainly fits with the aforementioned computational model of TTS (44), the necessary role of hyperthermia is likely multifactorial, particularly in the maintenance of a hypercateholaminergic state as would be present in human TTS precipitated by a stressful event.

### Clinical perspective

We have shown that hyperthermia is essential for the induction of TTS in this isoproterenol-induced model, and therefore it may be prudent that patients at risk of TTS are monitored for pyrexia and anti-pyretics are prescribed with a lower threshold. Furthermore, early echocardiographic imaging in patients with severe hyperthermic states, such as malignant hyperthermia or ICU admissions for sepsis, could reveal cardiac dysfunction, and guide clinical care. Nevertheless, the role of hyperthermia in human TTS is currently unknown, and as we were able to remove the hyperthermic state within 60 min of the induction agent being given (equivalent to the emotional stressor in humans), we cannot conclude that a reduction in body temperature *per se* would be of use in the management of the acute syndrome.

### Summary

In summary, we have shown that hyperthermia is essential in the induction of TTS in an isoproterenol-induced rat model, and the removal of hyperthermia around the time of the development of apical dysfunction does not attenuate or reverse it. Isolated cardiomyocyte studies showed that, at least in the unloaded state, baseline contractility, or isoproterenol-induced inotropic changes were not affected by hyperthermia. Further studies are needed to assess the role of hyperthermia in patients and, if so, whether this can be manipulated to improve patient outcome.

## Data availability statement

The raw data supporting the conclusions of this article will be made available by the authors, without undue reservation.

## Ethics statement

The animal study was reviewed and approved by Central Animal Welfare and Ethical Review Board, Imperial College London Animal Ethics Committee, Gothenburg University.

## Author contributions

MT, BR, and PW performed the experiments and analysis of data in this manuscript. MT wrote the initial draft of the manuscript, with contributions from PW, SH, and EO for the final draft. AL, EO, and SH supervised the research and had overall responsibility for the project. All authors contributed to the design of the experiments and analysis and interpretation of data. All authors contributed to the article and approved the submitted version.

## Funding

The study was financed by grants from the Swedish state under the agreement between the Swedish government and the county councils (the ALF-agreement), the Swedish Heart-Lung Foundation and the Swedish Scientific Council; by RG/17/13/33173, PG/17/3/32722; RG/17/6/32944; RG/12/18/30088; Medical Research Council UKMR/L006855/1; the National Heart and Lung Institute Foundation and British Heart Foundation BHF FS/16/52/32259.

## Conflict of interest

The authors declare that the research was conducted in the absence of any commercial or financial relationships that could be construed as a potential conflict of interest.

## Publisher's note

All claims expressed in this article are solely those of the authors and do not necessarily represent those of their affiliated organizations, or those of the publisher, the editors and the reviewers. Any product that may be evaluated in this article, or claim that may be made by its manufacturer, is not guaranteed or endorsed by the publisher.
