# Corrigendum: Hyperthermia as a trigger for Takotsubo syndrome in a rat model

**DOI:** 10.3389/fcvm.2022.1021913

**Published:** 2022-09-02

**Authors:** Matthew H. Tranter, Bjorn Redfors, Peter T. Wright, Liam S. Couch, Alexander R. Lyon, Elmir Omerovic, Sian E. Harding

**Affiliations:** ^1^Faculty of Medicine, Imperial College London, Hammersmith Campus, National Heart and Lung Institute (NHLI), London, United Kingdom; ^2^Oriel College, University of Oxford, Oxford, United Kingdom; ^3^Department of Molecular and Clinical Medicine/Cardiology, Sahlgrenska Academy, University of Gothenburg, Gothenburg, Sweden; ^4^School of Life and Health Sciences, University of Roehampton, London, United Kingdom

**Keywords:** Takotsubo, stress, hyperthermia, catecholamine, isoprenaline

In the published article, there was an error in the **Funding** statement.

**Funding**

The study was financed by grants from the Swedish state under the agreement between the Swedish government and the county councils (the ALF-agreement), the Swedish Heart-Lung Foundation and the Swedish Scientific Council; by RG/17/13/33173, PG/17/3/32722; RG/17/6/32944; RG/12/18/30088 and Medical Research Council UKMR/L006855/1; and the National Heart and Lung Institute Foundation.

A studentship from the British Heart Foundation was omitted. The correct **Funding** statement appears below.

## Funding

The study was financed by grants from the Swedish state under the agreement between the Swedish government and the county councils (the ALF-agreement), the Swedish Heart-Lung Foundation and the Swedish Scientific Council; by RG/17/13/33173, PG/17/3/32722; RG/17/6/32944; RG/12/18/30088; Medical Research Council UKMR/L006855/1; the National Heart and Lung Institute Foundation and British Heart Foundation BHF FS/16/52/32259.

The authors apologize for this error and state that this does not change the scientific conclusions of the article in any way. The original article has been updated.

### Publisher's note

All claims expressed in this article are solely those of the authors and do not necessarily represent those of their affiliated organizations, or those of the publisher, the editors and the reviewers. Any product that may be evaluated in this article, or claim that may be made by its manufacturer, is not guaranteed or endorsed by the publisher.

