# Supplementary material for: Hyperthermia as a trigger for Takotsubo syndrome in a rat model
Source: Front Cardiovasc Med. 2022 Jul 26;9:869585. doi: 10.3389/fcvm.2022.869585 (PMC9360576; doi:10.3389/fcvm.2022.869585)
Supplement: Supplementary file 1 [file Table_1.DOCX]

**Supplementary video**

Representative B-mode ultrasound of apical dysfunction in an isoprenaline-induced animal model of Takotsubo syndrome.
