# Supplementary material for: Hyperthermia as a trigger for Takotsubo syndrome in a rat model
Source: Front Cardiovasc Med. 2022 Jul 26;9:869585. doi: 10.3389/fcvm.2022.869585 (PMC9360576; doi:10.3389/fcvm.2022.869585)
Supplement: Supplementary file 3 [file Data_Sheet_1.PDF]

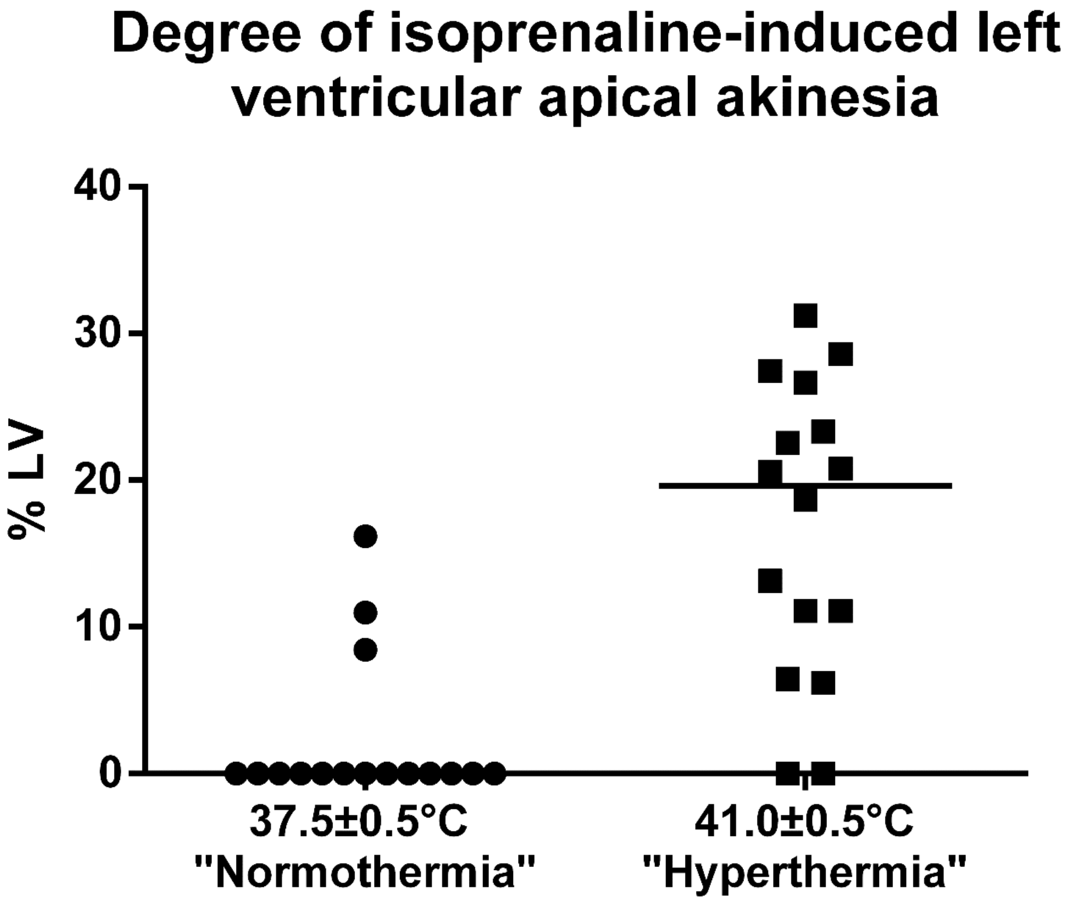

Supplementary figure 1: Degree of apical TCM-like cardiac dysfunction 90 min post 50mg/kg intraperitoneal isoprenaline. Rats randomized to hyperthermia developed significantly greater degree of apical TCM-like cardiac dysfunction compared to rats randomized to normothermia ($p<0.05$).

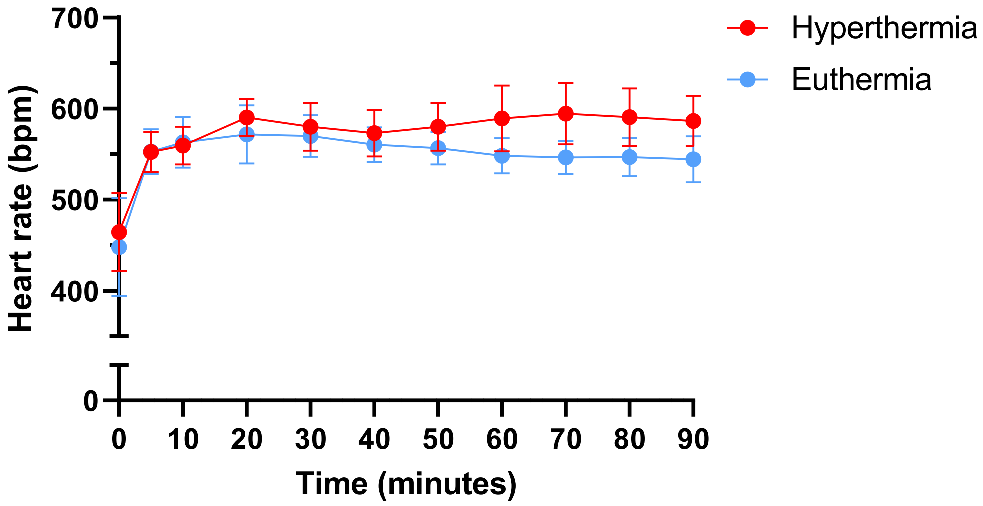

Supplementary figure 2: Heart rates for animals (n=6-8) with either euthermia or hyperthermia (contractility shown in figures 2 & 3). No statistical significance (P=0.59) was found between hyperthermic and euthermic, but a clear statistical difference was found across time-points (P<0.0001).
